# Residency in Long-Term Care Facilities: An Important Risk Factor for Respiratory Syncytial Virus Hospitalization

**DOI:** 10.1093/infdis/jiae424

**Published:** 2024-08-23

**Authors:** Angela R Branche, Ann R Falsey, Lyn Finelli, Edward E Walsh

**Affiliations:** Department of Medicine, Division of Infectious Diseases, University of Rochester, Rochester, New York, USA; Department of Medicine, Division of Infectious Diseases, University of Rochester, Rochester, New York, USA; Beacon Epidemiology Associates, Rose Valley, Pennsylvania, USA; Department of Medicine, Division of Infectious Diseases, University of Rochester, Rochester, New York, USA

**Keywords:** RSV hospitalization, RSV vaccine, long-term care facilities, RSV population-based incidence, Skilled Nursing Facilities

## Abstract

Older age and comorbid conditions increase risk for severe for respiratory syncytial virus (RSV). Skilled nursing facilities (SNFs) and assisted living (AL) facilities represent an intersection of risk factors. In a 3-year prospective study in Rochester, New York, we compared the population-based incidence of RSV-associated hospitalization for community-dwelling (CD), SNF, and AL adults aged ≥65 years. Their median ages were 76, 83 and 86 years, respectively, and dementia and congestive heart failure (CHF) were more prevalent among SNF and AL residents. The average annual incidences were 117 (95% confidence interval, 104–132), 440 (307–629) and 740 per 100 000 persons (523–1045), respectively, for CD, SNF, and AL adults aged ≥65 years, demonstrating a need for unequivocal RSV vaccine recommendations in SNF and AL residents.

## Teaser

In a 3-year prospective, population-based incidence study, respiratory syncytial virus hospitalization rates among adults aged ≥65 years were 3–9 and 3–4 times higher for those admitted from assisted living or skilled nursing facilities, respectively, compared with community-dwelling adults.

Respiratory syncytial virus (RSV) is a seasonal winter virus and a major cause of lower respiratory tract disease in older adults, resulting in approximately 60 000–160 000 hospitalizations and 6000–10 000 deaths in the United States annually among adults aged ≥65 years [1]. Research and development of interventions to prevent disease in older and at-risk adults have been underway for several decades and in May 2023, the Food and Drug Administration (FDA) approved 2 new RSV vaccines for prevention of RSV lower respiratory tract disease in adults aged ≥60 years. Following FDA approval, the US Centers for Disease Control and Prevention (CDC) initially recommended RSV vaccination for adults aged ≥60 years using shared clinical decision making [1] and further clarified guidance in June 2024 to recommend vaccinations for all adults aged ≥75 years and those aged 60–74 years at increased risk for severe RSV disease. Of critical importance, therefore, is to define clinical and demographic characteristics that increase the risk for severe RSV disease and identify the populations who will derive the greatest benefit from vaccine prevention.

Numerous studies have established older age as the most well-defined risk factor, such that the incidence of RSV-associated hospitalization increases significantly with each subsequent decade of life after 60 years [2–4]. Other risk factors include preexisting chronic medical conditions including chronic obstructive pulmonary disease (COPD), congestive heart failure (CHF) and diabetes, and underlying frailty [2, 5–7]. Residents of long-term care facilities (LTCFs), either skilled nursing facilities (SNFs) or assisted living (AL) facilities, represent an important intersection of these risk factors, encompassing older age, multimorbidity, and frailty. Although RSV was first recognized as an important pathogen in adult populations after being described as a cause of SNF outbreaks [8, 9], data on incidence in adults living in LTCFs remain scarce and derived primarily from passive surveillance or retrospective analyses of national claims database [4, 10, 11].

To capture the full burden of RSV infection among adults, we conducted a prospective, active surveillance study over 3 winters and calculated the population-based incidence of RSV-associated hospitalization in older adults and those living in LTCFs in Rochester, New York.

## METHODS

### RSV Surveillance and Data Collection

Surveillance for RSV infection in adults ≥18 years of age was conducted at 2 hospitals in Rochester, New York: Strong Memorial Hospital, of the University of Rochester Medical Center, and Rochester General Hospital of Rochester Regional Health System during 3 winters (15 October 2017 to 27 March 2020), as described elsewhere [2]. Adults with an acute respiratory infection (ARI), or exacerbations of CHF, COPD, or asthma preceded by ARI symptoms in the past 14 days, were screened for RSV by reverse-transcription polymerase chain reaction on admission, as described elsewhere [8]. Eligible patients were adults ≥18 years of age with laboratory-confirmed RSV, hospitalized for ≥24 hours, who resided in the predefined surveillance area (Monroe County, New York). Here we report an analysis of those aged ≥65 years.

Consent was obtained when possible and demographic and clinical data were extracted from the electronic medical record (EMR). Residence was verified using EMR and social work notes. Eligible RSV-infected patients who refused consent were included in incidence estimates, but no clinical or residence data could be extracted from the EMR. The study was approved by institutional review boards of each participating institution that permitted collection of limited demographics (age and sex) for nonconsented participants. The study concluded in late March 2020 at the beginning of the coronavirus disease 2019 pandemic, thus limiting the impact to premature termination of surveillance 1 month early in the 2019–2020 season.

### Statistical Analyses

Population-based RSV incidence rates and 95% confidence intervals were estimated for each of 3 seasons, for community-dwelling (CD) adults aged ≥65 years and 3 age subgroups: 65–74, 75–44, and ≥85 years. Age-based rates were compared with population-based incidence rates for adults aged ≥65 years living in SNFs and AL facilities. We did not report LTCF rates for each age subgroup due to the small number of cases for residents in each subgroup, which provide unstable estimates. For adults hospitalized with RSV who refused consent, the baseline living situation was imputed as CD.

Incidence for the CD cohort was estimated by dividing the number of hospitalized CD adults with laboratory-confirmed RSV infection by the adjusted 2018 US intercensal population estimate for the predefined catchment area. All incidence calculations were adjusted by the market share of Monroe County residents aged ≥65 years captured by the 2 hospitals, which was estimated to be 61% using 2016 Statewide Planning and Research Cooperative System (SPARCS) data (see Supplementary Methods).

The incidences for the LTCF (SNF or AL) cohorts were estimated by dividing the number of hospitalized adults residing in the respective LTCF type before admission with laboratory-confirmed RSV infection by the weekly Monroe County LTCF population bed census data [12]. In New York State, these data include the total numbers of beds available and occupied. We collected weekly bed census data from all LTCFs with zip codes in Monroe County and calculated the average bed occupancy during the surveillance period (October–April) as the denominator for incidence calculations. We then adjusted for age by multiplying occupied bed number by the national percentage of adults SNF or AL residents aged ≥65 years using 2020 CDC National Center for Health Statistics Biennial National Post-acute and Long term-Care Study estimates [13] (see Supplementary Methods).

## RESULTS

### Baseline Demographic and Clinical Characteristics

During the 3 winters, 7012 hospitalized adults meeting the case definition for ARI resided within Monroe County, New York, and were eligible for the incidence study. Of those, 89% were tested for RSV, and 532 RSV-infected hospitalized patients were identified [2]. In this analysis, we report findings on adults aged ≥65 years.

Of RSV-positive patients, 262 (49.2%) were CD adults and 62 (12%) were residents of SNFs or AL facilities ≥65 years of age. Detailed demographic and clinical data were available for 88% of all participants. CD adults were younger, with median ages of 76, 83, and 86 years respectively, for CD, SNF, and AL adults aged ≥65 years, and less likely to be female. Most patients were white (80%–90%) with 10%–16% identifying as black or African American and 0%–8% as Hispanic or Latinx (Table 1).

**Table 1.** Demographic and Clinical Characteristics of Older Adults Hospitalized With Respiratory Syncytial Virus in Rochester, New York, 2017–2020

Characteristic	Adults Aged ≥65 y Hospitalized With RSV, No. (%)^a^		
	CD Adults (n = 217^b^)	SNF Residents (n = 30)	AL Residents (n = 32)
Age, median (IQR)	76 (70–83)	83 (75–90)	86 (78–89)
Female sex	116 (53)	24 (80)	27 (84)
Race and ethnicity			
White	174 (80)	27 (90)	27 (84)
Black	23 (11)	3 (10)	5 (16)
Asian	5 (2)	0 (0)	0 (0)
Unknown	15 (7)	0 (0)	0 (0)
Hispanic	18 (8)	1 (3)	0 (0)
Chronic medical conditions			
Asthma	26 (12)	0 (0)	2 (6)
COPD	79 (36)	8 (27)	12 (38)
CAD	89 (41)	11 (37)	8 (25)
CHF	67 (31)	13 (43)	15 (47)
Diabetes mellitus	95 (44)	12 (40)	8 (25)
CKD	61 (28)	8 (27)	5 (16)
Immunocompromise^c^	30 (14)	2 (7)	1 (3)
Obesity (BMI ≥30^d^)	86 (39)	10 (33)	7 (22)
Dementia	20 (9)	9 (30)	10 (31)
CVA	27 (12)	4 (13)	7 (22)
No. of chronic conditions, median (IQR)	3 (2–4)	3 (1–4)	2 (2–3)
Clinical outcomes			
ICU care	27 (12)	11 (37)	1(3)
Mechanical ventilation	12 (6)	5 (17)	0 (0)
In-hospital death	11 (5)	3 (10)	0 (0)
Death within 6 mo	22 (10)	5 (17)	1 (3)
Length of hospital stay, median (IQR), d	5 (3–8)	9 (4–12)	5 (3–9)

Abbreviations: AL, assisted living; BMI, body mass index; CAD, coronary artery disease; CD, community-dwelling; CHF, congestive heart failure; CKD, chronic kidney disease; COPD, chronic obstructive pulmonary disease; CVA, cerebrovascular accident; ICU, intensive care unit; IQR, interquartile range; RSV, respiratory syncytial virus; SNF, skilled nursing facility.

^a^Data represent no. (%) of participants unless otherwise specified.

^b^Including only those who provided consent.

^c^Immunocompromise includes active chemotherapy, solid organ transplant, human immunodeficiency virus, or autoimmune or other condition requiring immunosuppressive therapy.

^d^BMI calculated as weight in kilograms divided by height in meters squared.

Most participants reported ≥1 comorbid condition before hospitalization, with a median of 3 conditions for CD adults and SNF residents aged ≥65 years and 2 conditions for AL residents (Table 1). The presence of specific chronic conditions of interest was similar among the 3 cohorts, except for CHF and dementia, which were more prevalent for SNF or AL residents than for CD adults (Table 1 and Supplementary Figure 1). COPD was identified in 27%–38%, and CHF in 31%–47% of each cohort. Other commonly reported conditions included diabetes (25%–44%), obesity (25%–47%) chronic kidney disease (16%–28%), and a history of cerebrovascular accident (12%–22%).

### Clinical Assessments and Outcomes

The median length of stay for participants in all 3 cohorts was 5 days (Table 1), but higher percentages of intensive care unit care and mechanical ventilation and higher in-hospital and 6-month all-cause mortality rates were noted for SNF adults aged ≥65 years compared with CD adults.

### Population-Based Incidence of RSV Hospitalization

**Figure 1.**
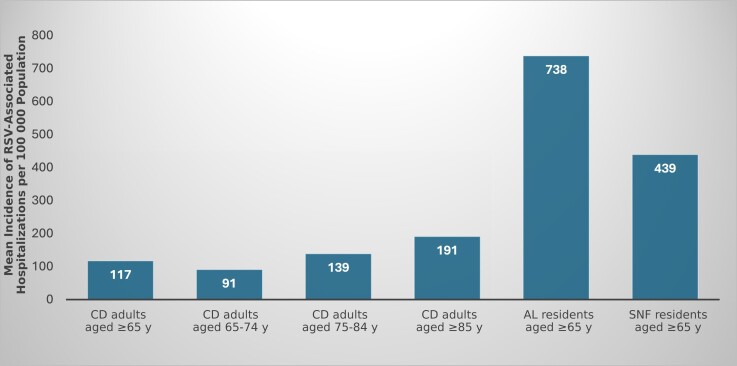
Mean annual incidence of respiratory syncytial virus (RSV)–associated hospitalization per 100 000 persons in Rochester, New York, over 3 winter seasons, 2017–2018, 2018–2019, and 2019–2020. The 95% confidence intervals for the point estimates are represented by vertical black bars. Abbreviations: AL, assisted living; CD, community-dwelling; SNF, skilled nursing facility.

The overall annual estimated incidence of hospitalization for CD adults aged ≥65 years was stable across 3 seasons and ranged from 111 to 127 per 100 000 population (Supplementary Table 1). The incidence in the CD cohort increased with age, with an average annual incidence across 3 seasons of 91, 139, and 191 per 100 000 population in CD adults 65–74, 75–84, and ≥85 years of age, respectively (Figure 1) Notably, the average annual incidences for adults aged ≥65 years living in LTCFs were higher (440/100 000 for SNFs and 740/100 000 for AL) than for even the highest age group of CD adults (aged ≥85 years). Observed rates were relatively stable in SNF residents (417–476/100 000), while rates for AL residents had a wider range (343–1018/100 000) (Figure 1 and Supplementary Table 1).

## DISCUSSION

In this prospective study we calculated population-based incidence of RSV-associated hospitalization among LTCF residents. We estimate rates of RSV-associated hospitalization 3–9 times higher for adults aged ≥65 years living in AL facilities and 3–4 times higher for those living in SNFs, compared with CD adults aged ≥65 years, and 2–5 and 2–3 times higher, respectively, compared with the oldest and presumably highest-risk age group of CD adults (aged ≥85 years). Most prior studies assessing the burden of RSV disease in LTCFs have described attack rates from seasonal outbreaks or relied on retrospective data from national claims database [10, 11]. These reports likely underestimated the incidence of cardiorespiratory hospitalization attributable to RSV, since RSV testing has not historically been routine clinical practice, and coding of RSV as the primary diagnosis for hospitalization remains inconsistent.

Interestingly, incidence rates for AL residents were higher than those in SNF residents. This seems counterintuitive but may reflect higher exposure risk for adults in AL facilities, who generally have more opportunities to engage in the wider community, or goals of care limitations that preclude hospitalization in the SNF group. However, the increased rates of RSV-associated hospitalization we observed for LTCF residents compared with CD adults, even at the highest CD age strata, highlight the need to consider LTCF residency an additional risk to other known risk factors. Notably, 25% of the adults in the SNF and AL cohorts from the original, prospective incidence study were <65 years old and exhibit many of the same characteristics of frailty that characterize the older residents. Consequently, their risk for RSV-associated hospitalization may be the same. These younger LTCF residents were not included in our incidence calculations.

The reasons behind the increased hospitalization in LTCF populations compared with CD older adults likely combine increased rates of infection due to congregate settings and increased risk for severe disease due to comorbid conditions and frailty [2, 4]. In our study, the only clear distinctions related to chronic medical conditions between the LTCF and CD cohorts were higher prevalence of CHF and dementia in the LTCF cohorts. This was similar to findings of a prospective RSV surveillance study of 1251 CD and 664 LTCF adults, where both incidence and attack rates were higher in the LTCF cohort, as were the prevalences of diabetes, COPD, cardiac, and renal disorders [11]. However, in contrast to that report, we did note worse clinical outcomes for adults aged ≥65 years admitted from an SNF with higher rates of intensive care unit care and mechanical ventilation and a slightly higher mortality rate compared with CD adults aged ≥65 years, which might be a surrogate measure of preexisting frailty. Moreover, our group has previously reported that functional decline after RSV hospitalization was significantly worse and more long term for adults admitted from SNFs [6]. Notably, though rates of RSV-associated hospitalization were highest in the AL cohort, severe clinical outcomes after hospitalization were better than in SNF residents and CD adults, which may reflect less frailty in the AL population at baseline.

Our study has a few important limitations. These results are from 2 health systems in Rochester, New York, and may not be generalizable to other geographic regions. There were 63 RSV-infected patients who declined to participate in the study. Although we collected basic demographics for these patients, an assumption was made that they were community dwelling. This may have resulted in underestimation of the SNF and AL incidence rates but would not change the overall findings of markedly higher rates in LTCF compared with CD adults. Second, population estimates of the ages of LTCF residents, used to adjust our incidence calculations, were based on both state and national data, which might not accurately represent our local LTCF population. Finally, we lacked data on goals of care for LTCF residents that may have limited hospitalization and affected SNF hospitalization rates.

However, to the best of our knowledge, our data represent the only prospective study comparing population-based estimates of incidence of RSV-associated hospitalization for CD, AL, and SNF adults aged ≥65 years, clearly demonstrating increased risk for severe clinical outcomes in the latter 2 populations. Further study is needed to assess whether our findings will be replicated in other geographic locations, but these data suggest a need for strong vaccine recommendations to prevent RSV disease in this vulnerable population.

## Supplementary Data

Supplementary materials are available at *The Journal of Infectious Diseases* online (http://jid.oxfordjournals.org/). Supplementary materials consist of data provided by the author that are published to benefit the reader. The posted materials are not copyedited. The contents of all supplementary data are the sole responsibility of the authors. Questions or messages regarding errors should be addressed to the author.

## Supplementary Material

jiae424_Supplementary_Data

## Notes

***Acknowledgments.*** The authors acknowledge Mary Criddle, Maryanne Formica, Sharon Moorehead, and Michael Peasley for their contributions to this study.

***Financial support*.** This work was supported by Merck Sharp & Dohme, a subsidiary of Merck & Co.
