# Supplementary material for: Residency in Long-Term Care Facilities: An Important Risk Factor for Respiratory Syncytial Virus Hospitalization
Source: J Infect Dis. 2024 Aug 23;230(5):e1007–11. doi: 10.1093/infdis/jiae424 (PMC11565855; doi:10.1093/infdis/jiae424)
Supplement: jiae424_Supplementary_Data [file jiae424_supplementary_data.docx]

**Supplemental Methods: Calculation of Incidence rates, with data sources for estimate adjustments**

Community Dwelling (CD) Population

Input for Numerator

A = No. RSV PCR+ community dwelling^a^ hospitalized adults > 65 years of age

Input for Denominator

B = No. Monroe County, NY residents age ≥ 65 years of age, and age subgroups^b^

C = Market Share % for the two hospitals^c^

**Annual Incidence**_CD_= A /(B x C), expressed as per 100,000 with 95% CI

Long Term Care Facility (LTCF) Population

Input for Numerator

D = No. Monroe County, NY residents ≥ 65 years of age residing in LTCF^a^

Input for Denominator

E = No. LTCF beds in Monroe County, NY^d^

F = Estimated % of LTCF beds occupied by persons ≥ 65 years of age^e^

**Annual Incidence_LTCF_** = D/(E x F x C), expressed as per 100,000 with 95% CI

^a^ Electronic Medical Record address indicative of community dwelling or LTCF residence, confirmed by review of social work admission and discharge notes.

^b^ US Census data for intercensal populations by age group for Monroe County, NY

^c^  estimate derived from SPARKS data for the two hospitals (URMC SMH and RGH), verified by using data for specific respiratory discharge diagnoses (e.g., COPD exacerbation)

^d^ New York State Department of Health weekly census data for all LTCF residents during the months of surveillance

^e^ US CDC NPALS estimates of % of LTCF residents (SNF and Assisted Living) ≥ 65 years of age occupying LTCF beds

**Supplemental Table 1: Annual Incidence Rates of RSV-Associated Hospitalization by RSV Season**

|  | **October 2017 – April 2018**  **Rate (95% CI)** | **October 2018 – April 2019**  **Rate (95% CI)** | **October 2019 – April 2020**  **Rate (95% CI)** | **All Seasons**  **Rate (95% CI)** |
| --- | --- | --- | --- | --- |
| **CD Adults > 65 years** | 115 (92-141) | 111 (90-138) | 127 (103-155) | 117 (104-132) |
| **CD Adults 65-74 years** | 99 (73-133) | 76 (54-106) | 99 (73-133) | 91 (76-109) |
| **CD Adults 75-84 years** | 126 (87-184) | 150 (106-212) | 141 (98-201) | 139 (113-171) |
| **CD Adults ≥ 85 years** | 158 (96-264) | 190 (120-302) | 224 (146-343) | 191 (146-249) |
| **Assisted Living > 65 years** | 856 (487-1504) | 1018 (615-1684) | 343 (143-823) | 738 (523-1043) |
| **SNF > 65 years** | 476 (264-859) | 417 (217-800) | 424 (228-787) | 439 (307-628) |

**Supplemental Figure 1: Prevalence of Chronic Medical Conditions in Older Adults Hospitalized with RSV in Rochester, NY 2017-2020**
